# Epigenetic ageing is accelerated in drug-resistant epilepsy and dynamically modulated during ketogenic diet therapy

**DOI:** 10.1016/j.ebiom.2026.106449

**Published:** 2026-08-22

**Authors:** Kristina Gervin, Magnhild Kverneland, Karl Otto Nakken, Knut Rudi, Per Ole Iversen, Kaja Kristine Selmer

**Affiliations:** a Division of Clinical Neuroscience, Department of Research and Innovation, Oslo University Hospital, Oslo, Norway; b Division of Clinical Neuroscience, National Centre for Epilepsy, Oslo University Hospital, Oslo, Norway; c Faculty of Chemistry, Biotechnology and Food Science, Norwegian University of Life Sciences, Ås, Norway; d Department of Haematology, Oslo University Hospital, Oslo, Norway; e Department of Nutrition, University of Oslo, Oslo, Norway

**Keywords:** Epigenetic age, DunedinPACE, Ketogenic diet, Treatment refractory epilepsy, Health-related quality of life

## Abstract

**Background:**

The ketogenic diet (KD) is an established treatment for drug-resistant epilepsy. Beyond seizure reduction, many patients report improved energy, cognition, and well-being. DNA methylation (DNAm)–based epigenetic clocks provide molecular indices of biological ageing and may capture short-term adaptations induced by dietary therapy.

**Methods:**

Fifty-eight adults with drug-resistant epilepsy completed a 12-week modified KD intervention with blood sampling at baseline, four weeks, and 12 weeks. Whole-blood DNAm was profiled using the Illumina EPIC array. Epigenetic ageing was estimated using cumulative clocks (Horvath, Hannum, Levine) and the rate-based DunedinPACE clock. Longitudinal changes were assessed using linear mixed-effects models. DunedinPACE trajectories were clustered using k-means. Associations with β-hydroxybutyrate, seizure frequency, body weight, and health-related quality of life were assessed using correlations and between-cluster comparisons.

**Findings:**

At baseline, participants showed substantial epigenetic age acceleration on cumulative clocks. Cumulative age acceleration did not change during the intervention. In contrast, DunedinPACE revealed two marked ageing-rate trajectories: an initial increase followed by slowing (“up–down”) and an initial slowing followed by rebound (“down–up”). Mean ageing rate did not change at the group level (p = 0.18). However, the “up–down” cluster exhibited significantly greater improvement in quality of life compared with the “down–up” cluster (ΔQOLIE 18.8 versus 4.7; p = 0.007) independent of ketosis, seizure reduction, or weight change.

**Interpretation:**

Drug-resistant epilepsy is associated with increased cumulative epigenetic ageing. While cumulative age remained stable during modified KD therapy, dynamic ageing-rate patterns were linked to patient-reported benefit, suggesting that rate-based DNAm clocks capture short-term systemic adaptation to metabolic treatment.

**Funding:**

This study was funded by the 10.13039/100009471Dam Foundation, the Norwegian Epilepsy Association’s Research Fund, the 10.13039/501100009708Novo Nordisk Foundation, and the 10.13039/100031364National Advisory Unit on Rare Disorders.

**Research in context**

**Evidence before this study**

Drug-resistant epilepsy is associated with substantial systemic comorbidity and increased long-term health burden, suggesting involvement of chronic inflammatory, metabolic, and stress-related pathways. DNAm-based epigenetic clocks provide quantitative biomarkers of biological ageing that integrate multisystem physiological signals and predict morbidity and mortality in population studies. Emerging data suggest that epilepsy may be accompanied by accelerated biological ageing, but systematic characterisation in well-phenotyped clinical cohorts is limited. Lifestyle and metabolic interventions, including KD, have been shown to influence DNAm-based ageing measures in other populations, yet whether such biomarkers capture systemic disease burden or short-term physiological adaptation during epilepsy treatment has not been explored. Rate-based measures of biological ageing have not been applied to repeated-measures intervention studies in adults with drug-resistant epilepsy.

**Added value of this study**

We combined longitudinal DNAm profiling with clinical and patient-reported outcomes during 12 weeks of modified KD therapy in adults with drug-resistant epilepsy. At baseline, participants exhibited epigenetic age acceleration using cumulative clocks, consistent with elevated systemic biological ageing burden. During treatment, cumulative age measures remained stable, whereas the rate-based DunedinPACE clock revealed marked inter-individual heterogeneity in short-term ageing trajectories. Importantly, trajectory pattern rather than absolute ageing rate was associated with improvement in patient-reported quality of life. This finding is clinically relevant because many individuals undergoing KD therapy report improved well-being independent of seizure reduction, suggesting broader systemic adaptation beyond anticonvulsant effects.

**Implications of all the available evidence**

Taken together with evidence that epigenetic ageing biomarkers reflect multisystem health and are responsive to metabolic change, our results suggest that measures of epigenetic ageing may capture systemic physiological alterations associated with drug-resistant epilepsy. Rate-based DNAm clocks could be useful for monitoring short-term systemic responses to KD therapy and for relating molecular changes to clinical outcomes and patient-reported quality of life.

## Introduction

KD is a well-established non-pharmacological therapy for drug-resistant epilepsy in children and has also shown evidence of efficacy in adults.^1^^,^^2^ By inducing a state of nutritional ketosis through high-fat, low-carbohydrate intake, KD is believed to provide an alternative energy substrate for the brain and to exert broad neuroprotective and metabolic effects.^3^ While seizure reduction usually remains the primary therapeutic goal, many patients report marked improvements in cognition, mood and overall well-being even in the absence of seizure remission.^4^^,^^5^ These systemic benefits suggest that KD influences not only neuronal excitability, but also fundamental physiological processes related to mitochondrial function, inflammation, and cellular resilience.^6^^,^^7^ However, the molecular mechanisms underlying these wider therapeutic effects remain poorly characterised.

DNAm-based epigenetic clocks have emerged as molecular indices of biological ageing and systemic physiological state. These clocks integrate DNAm patterns across hundreds of CpG sites, and different generations of clocks capture distinct biological dimensions of ageing. First-generation clocks (e.g., Horvath,^8^ Hannum^9^) are trained to predict chronological age, reflecting cumulative molecular ageing across tissues. Second-generation clocks, such as Levine, integrate DNAm with clinical biomarkers of mortality risk, offering improved prediction of disease and health span.^10^ Third-generation clocks, including DunedinPACE,^11^ quantify the pace or rate of biological ageing, derived from longitudinal multi-system biomarker change.

Accelerated epigenetic age, i.e., when DNAm-predicted age exceeds chronological age, has been linked to chronic inflammation, metabolic dysfunction and increased morbidity and mortality.^12^^,^^13^ Conversely, lifestyle and pharmacological interventions have been shown to slow biological ageing, including changes captured by epigenetic clocks.^14^ Diet appears to be a modulator of epigenetic ageing, as also suggested by a recent preprint.^15^ Recently, a very-low-calorie ketogenic diet was shown to slow DNAm-based biological age in individuals with obesity, consistent with metabolic reprogramming through nutritional ketosis.^16^ Similar effects have been reported following caloric restriction^17^ and in multifactorial lifestyle interventions combining diet, vitamin D, omega-3 fatty acids and exercise.^14^ Consistently, population-based analyses link poor diet quality to accelerated epigenetic ageing^18^ and show robust associations between diet and DNAm-based ageing markers.^19^

Individuals with epilepsy may exhibit features of accelerated biological ageing due to chronic neuroinflammation, oxidative stress, frequent comorbidities and long-term exposure to anti-seizure medications.^20^^,^^21^ However, evidence on epigenetic ageing in epilepsy is limited. Whether and how DNAm-based biological ageing measures relate to metabolic interventions such as ketogenic diet therapy, remains unknown.

KD induces rapid alterations in mitochondrial metabolism, inflammatory signalling, and redox homoeostasis, processes central to the hallmarks of biological ageing and linked to underlying epigenetic patterns.^7^^,^^22^^,^^23^ We therefore hypothesised that treatment with modified KD may influence the biological ageing rate. By applying complementary epigenetic clocks capturing distinct dimensions of ageing biology, including cumulative molecular ageing and the current pace of biological ageing, we aimed to: 1) characterise baseline ageing burden in adults with epilepsy, and 2) assess short-term physiological ageing dynamics during KD therapy. Evaluating these molecular changes alongside clinical and patient-reported outcomes may help clarify the broader systemic impact of KD beyond seizure reduction.

## Methods

### Study design and characteristics

This study included 58 adults with drug-resistant focal or generalised epilepsy recruited at the National Centre for Epilepsy (Oslo University Hospital) with available DNAm data. The cohort consisted of patients with focal epilepsy^24^ and generalised epilepsy,^2^ and inclusion criteria and dietary procedures have been described previously.^2^^,^^24^^,^^25^ In brief, participants were men and women ≥16 years with epilepsy, a BMI > 18.5 kg/m^2^, ≥three observable seizures per month despite trials of at least three antiseizure drugs, and who were motivated and considered able to follow the dietary treatment. They were excluded if they had recent status epilepticus, recent vagus nerve stimulator implantation, previous KD use, psychogenic non-epileptic seizures, pregnancy, or comorbidities contraindicating the diet. Participants followed a modified ketogenic diet for 12 weeks after a 12-week baseline period. Carbohydrate intake was restricted to <16 g/day. High-fat foods were encouraged. Venous blood was collected at baseline, four weeks, and 12 weeks after overnight food and drug fast for biochemical and DNAm analyses. The morning blood concentration of 3-hydroxybutyrate was assessed by a fingerprick blood sample using ABBOTT Freestyle Optium Blood Glucose Test Strips and Precision Xtra Blood Ketone Test Strips (Abbott). The Quality of Life in Epilepsy Inventory-89 (QOLIE-89) patient-reported questionnaire was completed immediately before starting and after completing the 12-week intervention period.^26^ Years with epilepsy was defined as years since clinician-confirmed epilepsy diagnosis. Weekly seizure frequency was derived from daily seizure diaries and analysed as percentage change from baseline. For the present analyses, seizure diary data were summarised as aggregated counts. The diary dataset did not include harmonised time-of-day information suitable for circadian analyses. Concurrent anti-seizure treatment (drug and dose, and n. vagus stimulator) were kept unchanged throughout the study period.

### DNA methylation profiling and clock estimation

Whole-blood DNAm was quantified using the Illumina Infinium MethylationEPIC BeadChip (850 K) as previously described.^27^ Preprocessing followed our published pipeline and included background correction, dye-bias adjustment, and quantile normalisation.^27^ Briefly, probes with detection p > 0.01 in >5% of samples, cross-reactive or polymorphic probes, and probes located on sex chromosomes were removed. Samples failing standard QC criteria (call rate <95% or outlier signal intensity) were excluded prior to analysis. The final dataset consisted of 760,462 probes across 172 samples. Cell-type proportions (CD4^+^ T cells, CD8^+^ T cells, B cells, NK cells, monocytes, and granulocytes) were estimated using the estimateCellCounts2 function in the FlowSorted.Blood.EPIC R package.^28^ To minimise technical batch effects in the longitudinal analyses, samples from the same participant (baseline, four weeks, and 12 weeks) were processed on the same BeadChip. Within each chip, samples were randomly distributed across Sentrix positions to reduce potential positional bias.

Epigenetic age and ageing rate were estimated using four established DNAm-based clocks: 1) the Horvath clock,^8^ 2) the Hannum clock,^9^ 3) the Levine clock integrating physiological and mortality-associated markers^10^ and 4) the DunedinPACE clock quantifying the current rate of biological ageing.^11^ The cumulative clocks were computed using the *methylclock* R package.^29^ DunedinPACE scores (v1.2.0) were derived using the *PACEProjector* function and represents the rate of biological ageing, where values > 1 indicate accelerated and values < 1 indicate slowed ageing. DunedinPACE scores were used as the primary outcome in all subsequent analyses of within-individual change and trajectory clustering.

### Statistical analyses

Statistical analyses addressed three components: (i) baseline epigenetic age acceleration and longitudinal stability of cumulative clocks, (ii) group-level and individual-level changes in epigenetic ageing rate, and (iii) associations between ageing-rate dynamics and clinical or patient-reported outcomes.

Baseline epigenetic age acceleration (DNAm age - chronological age) was calculated for the Horvath, Hannum, and Levine clocks. Concordance between DNAm-predicted age and chronological age was assessed using Pearson and Spearman correlation coefficients. Intraclass correlation coefficients (ICCs) across baseline, four weeks, and 12 weeks were computed from random-intercept mixed-effects linear models (participant as a random intercept) to quantify longitudinal stability of the cumulative clocks. Baseline associations between epigenetic age acceleration and clinical variables were assessed using linear models testing age acceleration against baseline seizure frequency (mean seizures/week from diaries), sex, and epilepsy duration.

Longitudinal changes in DunedinPACE were tested using linear mixed-effects models implemented in the *lme*4 package,^30^ with timepoint as a fixed effect, estimated cell types as covariates and participant as a random intercept to account for repeated measures. For clinical variables (β-hydroxybutyrate, seizure frequency, body weight, and QOLIE-89), change scores were computed as Δ = value for 12 weeks - baseline.

To explore heterogeneity in ageing-rate responses during modified KD therapy, we applied two complementary k-means clustering strategies to repeated DunedinPACE measures at baseline, four weeks, and 12 weeks. First, *level-based* clustering was performed on the three absolute DunedinPACE values (baseline, four weeks, 12 weeks) after z-standardisation across participants, with the number of clusters prespecified to *k* = 2, to separate individuals with overall lower versus higher DunedinPACE profiles. Second, *shape-based* clustering focused on temporal response by clustering two change features: ΔPACE (four weeks − baseline) and ΔPACE (12 weeks − four weeks). These delta features were z-standardised across participants and clustered using k-means with *k* = 2, yielding two dominant trajectory-response patterns (“down–up” versus “up–down”). To assess the choice of cluster number for the *shape-based* clustering, we compared *k* = 1–4 using within-cluster sum of squares (elbow) and mean silhouette width; these diagnostics supported *k* = 2 (Supplementary Fig. S1). Cluster assignments were subsequently used for between-cluster comparisons of clinical and patient-reported outcomes.

Associations between ΔPACE and clinical change measures were assessed using Pearson correlation coefficients. Clinical change measures included change in β-hydroxybutyrate (ΔBHB; continuous marker of ketosis), change in total QOLIE-89 score (ΔQOLIE), percentage change in weekly seizure frequency from baseline, and change in body weight (ΔWeight). Change scores were calculated as Δ = value at 12 weeks − baseline. Spearman rank correlations were computed as sensitivity analyses. Benjamini–Hochberg FDR correction was applied to sets of related tests (Supplementary Tables S1–S3), with FDR < 0.05 considered statistically significant.

Between-cluster differences in clinical outcomes were evaluated using Welch’s t-tests to account for unequal variances and cluster sizes. Findings were validated using Wilcoxon rank-sum tests and non-parametric bootstrap resampling (10,000 iterations) to generate bias-corrected and accelerated (BCa) 95% confidence intervals for the mean difference in ΔQoLIE.

### Ethics

The study was approved by the Regional Committee for Medical and Health Research Ethics, South-Eastern Norway (reference number 2010/2326). All participants provided written informed consent before enrolment. All procedures were conducted in accordance with the ethical approval and the Declaration of Helsinki. The randomised clinical study was registered at ClinicalTrials.gov (NCT01311440) on March 9, 2011.

### Role of funders

The funders provided financial support for the study but had no role in the design or conduct of the study, data collection, data analysis, data interpretation, writing of the manuscript, or the decision to submit the manuscript for publication. The authors were not precluded from accessing data in the study and accept responsibility for the decision to submit for publication.

## Results

### Participant characteristics

A total of 58 adults with drug-resistant epilepsy contributed DNAm data at baseline (n = 58), four (n = 56) and 12 (n = 48) weeks and were included in the epigenetic ageing analyses. Table 1 summarises demographic, clinical, metabolic, and DNAm-based ageing characteristics at study entry. Participants had a mean age of 35.7 years, 53.4% were women, and the cohort showed long-standing disease with a mean duration of epilepsy of 24.8 years. Baseline seizure burden was high (mean 22.1 seizures/week), and a mean quality of life scores (based on the QOLIE-89 tool) at 61.9 reflected substantial disease impact.

**Table 1:** Baseline clinical characteristics and epigenetic ageing measures of study participants.

Characteristics	Frequency
Number of participants	58
Age, years	35.7 (12.0)
Female sex, n (%)	31 (53.4)
Epilepsy classification, n (%)	
Focal	49 (85%)
Generalised	9 (15%)
Epilepsy aetiology, n (%)	
Structural	16 (27.6%)
Genetic	7 (12.1%)
Infectious	4 (6.9%)
Unknown	31 (53.4%)
Years with epilepsy	24.8 (12.4)
Baseline seizure frequency, mean/week	22.1 (60.8)
Intellectual disability, n (%)	21 (36%)
Employment, n (%)	
Paid	13 (22%)
Occupationally disabled	37 (64%)
Other	8 (14%)
Weight, kg	79.1 (18.2)
BMI, kg/m^2^	26.9 (5.6)
β-hydroxybutyrate, mmol/L	0.08 (0.11)
QOLIE-89 score	61.9 (15.1)
Anti-seizure medications	
Monotherapy, n (%)	15 (25.9)
Polytherapy, n (%)	37 (63.8)
At diet initiation, mean	2.1 (0.9)
Carbamazepine, n (%)	7 (12.1)
Zonisamide, n (%)	5 (8.6)
Levetiracetam, n (%)	14 (24.1)
Valproate, n (%)	15 (25.9)
Topiramate, n (%)	10 (17.2)
Oxcarbazepine, n (%)	16 (27.6)
Phenytoin, n (%)	3 (5.2)
Clobazam, n (%)	9 (15.5)
Lamotrigine, n (%)	12 (20.7)
Phenobarbital, n (%)	2 (3.4)
Lacosamide, n (%)	6 (10.3)
Pregabalin, n (%)	2 (3.4)
Clonazepam, n (%)	2 (3.4)
DNAm ageing measures	
Horvath DNAm age, years	45.1 (11.8)
Hannum DNAm age, years	52.3 (11.2)
Levine DNAm age, years	25.8 (13.5)
Horvath age acceleration, years	8.5 (5.7)
Hannum age acceleration, years	15.6 (5.1)
Levine age acceleration, years	−10.7 (7.4)
DunedinPACE	0.94 (0.11)

Data are mean (SD) unless otherwise indicated.

BMI = body mass index; DNAm = DNA methylation; QOLIE-89 = Quality of Life in Epilepsy Inventory-89; DunedinPACE = Dunedin Pace of Ageing methylation algorithm.

### Cumulative epigenetic age estimates across clocks

**Fig. 1:**
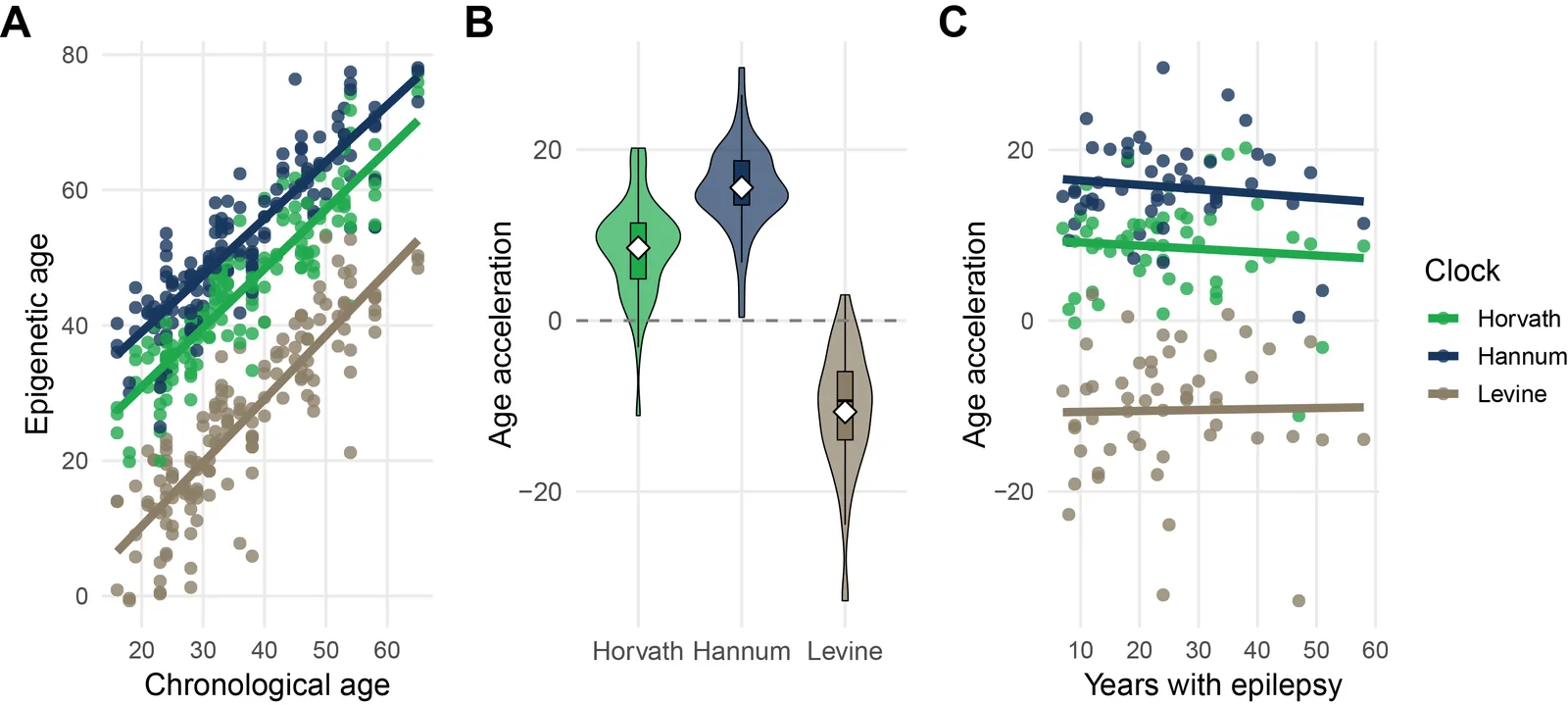
**Baseline characteristics of cumulative epigenetic clocks. A)** Scatterplots showing the correlation between chronological age and DNAm-predicted age for the Horvath, Hannum and Levine clocks. Each point represents an individual at baseline. All three clocks showed strong correlations with chronological age, confirming the expected biological signal in DNAm-based age estimates. Solid lines indicate linear regression fits. **B)** Distribution of epigenetic age acceleration at baseline for each clock. Violin plots show the full distribution, with embedded boxplots and white diamonds marking the interquartile range and mean, respectively. **C)** Scatterplots showing the association between disease duration and baseline epigenetic age acceleration across the clocks. Lines represent linear regression fit. No clock showed a significant association between disease duration and age acceleration, indicating that the elevated biological age observed in this cohort does not increase with longer epilepsy duration.

At baseline, DNAm-based age estimates from the Horvath, Hannum and Levine clocks showed strong correlations with chronological age (r = 0.87–0.90, p < 2.2 × 10^−16^, Fig. 1A). Spearman correlations were similar (ρ = 0.87–0.90, p < 2.2 × 10^−16^). Baseline epigenetic age acceleration (DNAm age − chronological age) differed across clocks, with mean values of +8.5 ± 5.7 years for Horvath, +15.6 ± 5.2 years for Hannum and −10.7 ± 5.1 years for Levine (Fig. 1B). The negative offset observed for the Levine clock likely reflects that this clock was trained to approximate a DNAm-based phenotypic age derived from a composite of clinical biomarkers predictive of mortality risk, rather than to predict biological age, and should not be interpreted as true deceleration of biological ageing. These findings indicate that adults with drug-resistant epilepsy exhibit substantial DNAm-derived epigenetic age acceleration relative to chronological age, consistent with an elevated cumulative molecular ageing burden. To evaluate longitudinal stability, we also calculated ICCs across baseline, four weeks and 12 weeks. ICCs were moderate for all three clocks (Horvath = 0.624; Hannum = 0.576; Levine = 0.520), indicating that most variance arose between individuals rather than from within-individual fluctuation over time. These values are consistent with the known design of cumulative clocks as relatively stable long-term ageing markers.

To examine whether the observed epigenetic age acceleration reflected cumulative years lived with epilepsy, we tested the association between age acceleration and disease duration at baseline. Across all three clocks, disease duration showed no significant correlation with age acceleration (Horvath: r = −0.08, p = 0.56; Hannum: r = −0.12, p = 0.39; Levine: r = 0.02, p = 0.89, Fig. 1C, Supplementary Table S1). Also, linear regression models adjusting for chronological age likewise demonstrated no evidence that longer epilepsy duration predicted greater age acceleration (all p > 0.64). Likewise, baseline seizure frequency showed no evidence of association with age acceleration (all p ≥ 0.46; Supplementary Table S1). Male participants showed higher Horvath age acceleration (β = 4.56 years; FDR = 0.010), whereas no sex differences were observed for Hannum or Levine (both p ≥ 0.16; Supplementary Table S1).

Longitudinal modelling using linear mixed-effects models showed no significant effect of time for any cumulative clock (Horvath FDR = 0.64; Hannum FDR = 0.081; Levine FDR = 0.98), confirming the absence of systematic change during the 12-week intervention. We next assessed within-individual change from baseline to four and 12 weeks. After FDR correction, none of the clocks showed significant change at either interval (Supplementary Table S2). Although the Hannum clock showed a small increase at 12 weeks (+2.06 years, FDR = 0.045), this was not replicated in the other clocks.

### Short-term dynamic changes in biological ageing rate

We next applied the DunedinPACE clock, which estimates the current physiological rate of biological ageing rather than cumulative biological age. At baseline, DunedinPACE averaged 0.94 ± 0.11 (range: 0.70–1.22). Because DunedinPACE is scaled such that 1.0 reflects the reference pace of ageing, this corresponds to a slightly slower pace of ageing at baseline.

**Fig. 2:**
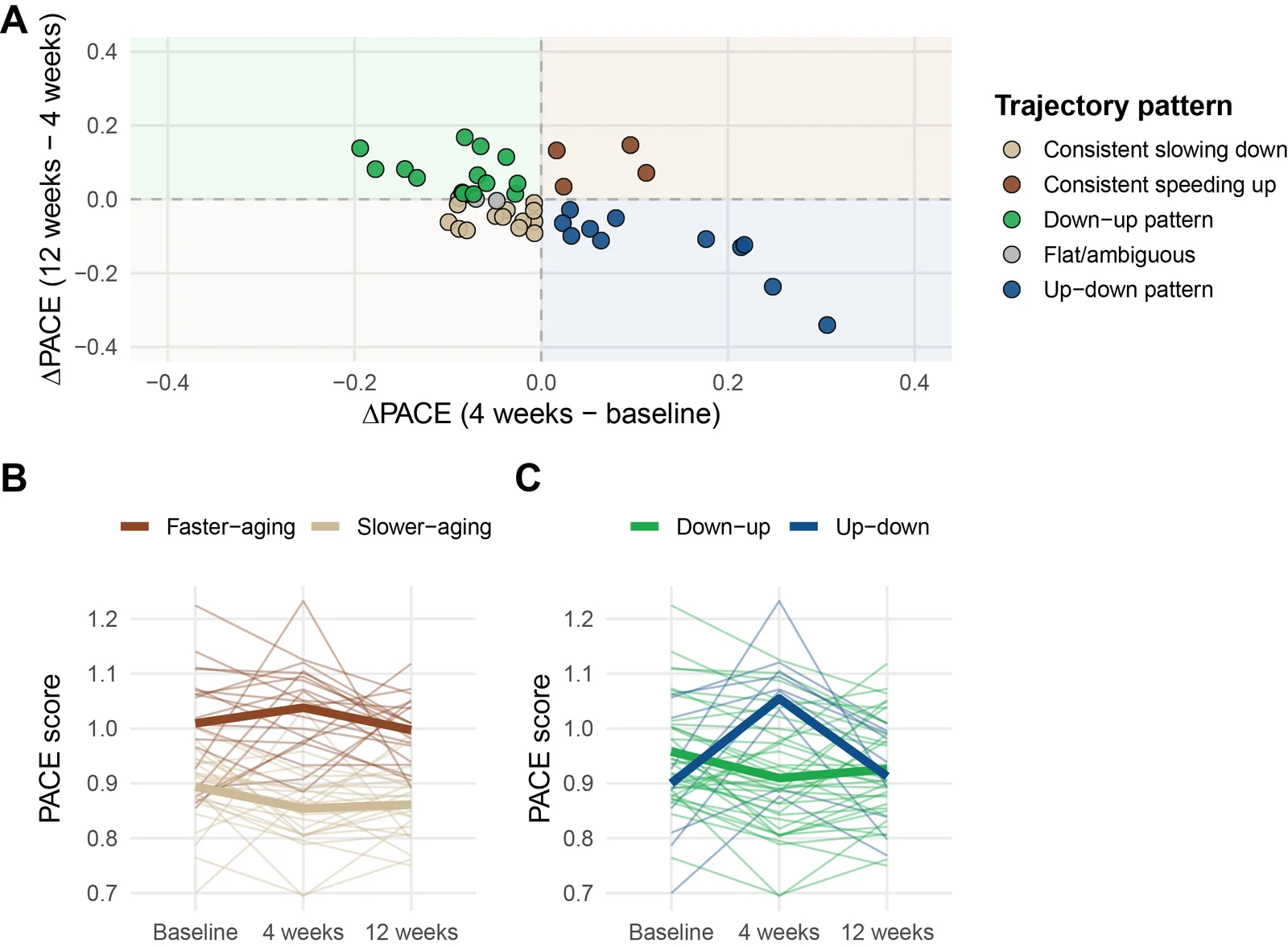
**Heterogeneity in DunedinPACE trajectories during ketogenic diet therapy. A)** Scatterplot showing individual short-term changes in the biological ageing rate. Each point represents one participant, positioned by their change in DunedinPACE between baseline and four weeks (x-axis) and between four and 12 weeks (y-axis). Dashed lines indicate no change; colours denote trajectory categories: consistent slowing down (beige), consistent speeding up (brown), and transient patterns (green and blue). **B)** Clustering by absolute DunedinPACE levels identifies participants with overall slower versus faster biological ageing rates (“slow” and “fast” agers). **C)** Clustering based on temporal change distinguishes two dominant response patterns; an initial decrease followed by rebound (“down–up”) and an initial increase followed by subsequent decline (“up–down”). Each thin line represents an individual participant, and thick lines indicate the cluster-specific mean trajectory. DunedinPACE values < 1.0 reflect a DNAm-based ageing rate slower than one chronological year per year, whereas values > 1.0 indicate accelerated ageing.

A linear mixed-effects model showed no significant group-level change in DunedinPACE across the 12-week intervention (F = 1.77, p = 0.18). Despite the stable group mean, individual trajectories varied markedly, with some participants exhibiting transient slowing and others showing temporary acceleration of ageing rate (Fig. 2A). Trajectory clustering was restricted to participants with DunedinPACE available at all three timepoints (complete cases). This inter-individual variability motivated further exploration of whether distinct physiological response patterns might exist. To characterise this heterogeneity, we applied two complementary clustering approaches to the individual DunedinPACE trajectories. First, level-based clustering grouped participants into slower-ageing (n = 25) and faster-ageing (n = 21) individuals based on absolute DunedinPACE values (Fig. 2B). These clusters describe stable between-individual differences in baseline physiological ageing rate. These were not expected to capture short-term dynamic responses but provide a reference against which dynamic changes can be interpreted.

Second, to identify dynamic physiological response patterns, we clustered individuals based on the shape of their within-person DunedinPACE trajectories across the three timepoints. Two dominant temporal patterns emerged: an initial decrease followed by partial rebound (“down–up”, n = 37) and an initial increase followed by later reduction (“up–down”, n = 9) (Fig. 2C). These trajectory-based clusters reflect short-term modulation of biological ageing rate during modified KD and formed the basis for subsequent analyses linking physiological adaptability to clinical and quality-of-life outcomes.

### Association of ageing rate with clinical outcomes

To examine whether changes in epigenetic ageing rate were related to changes in clinical metabolic or patient-reported outcomes during ketogenic diet therapy, we evaluated associations between ΔPACE and four key outcomes: change in β-hydroxybutyrate (ΔBHB), percentage change in seizure frequency (ΔSeizure), body weight change (ΔWeight) and change in quality of life (ΔQoLIE).

First, we analysed continuous associations at the individual level. Pearson correlations between ΔPACE and the four clinical variables were small and non-significant (all |r| < 0.3, p > 0.25; Supplementary Table S3), indicating that the magnitude of the ageing-rate change itself was not directly linked to metabolic ketosis, seizure reduction, weight loss or quality of life. Spearman rank correlations were computed as sensitivity analyses yielding consistent results.

**Fig. 3:**
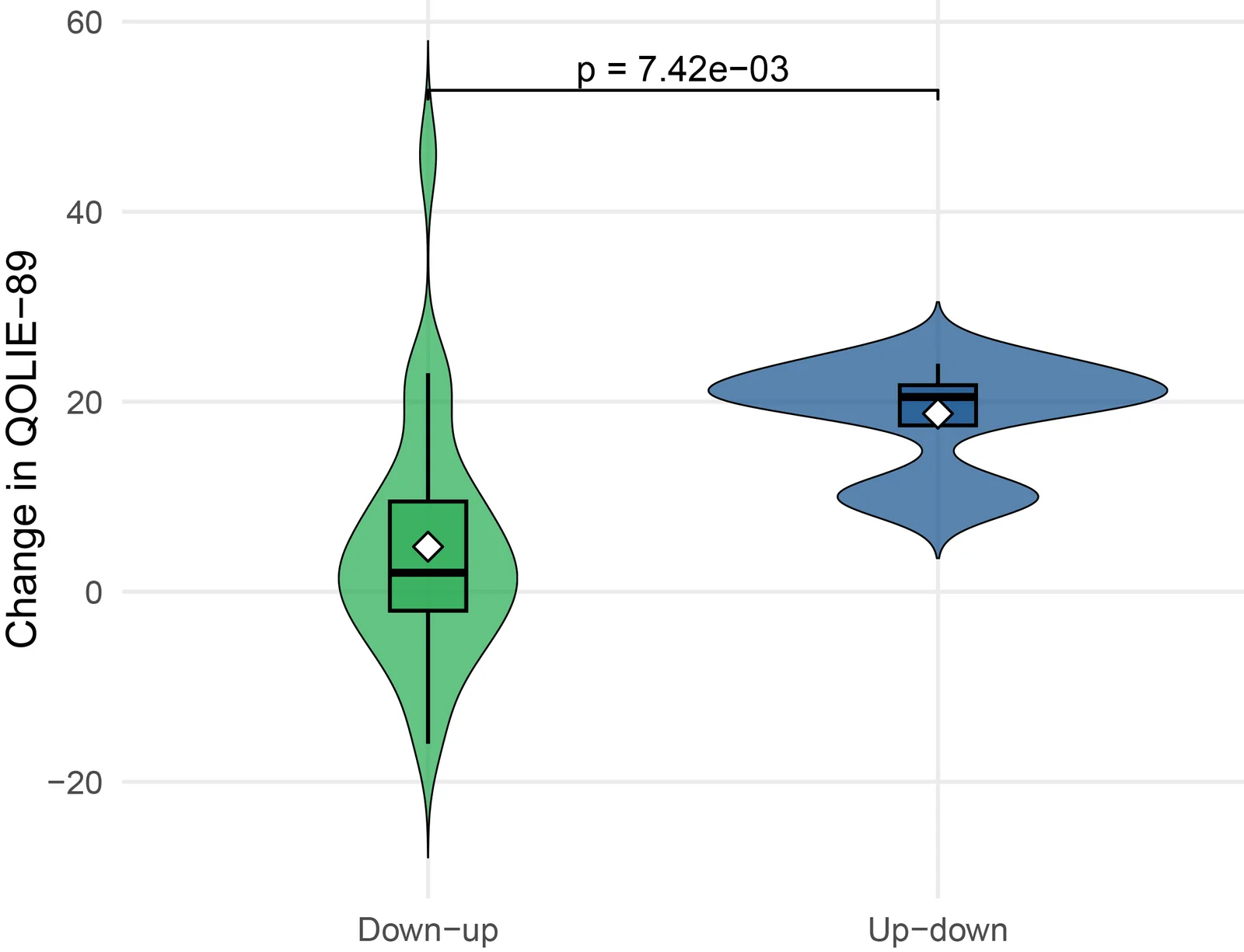
**Improvement in quality of life by DunedinPACE trajectory pattern during KD treatment.** Violin plot showing change in QoLIE-89 across the two trajectory classes identified in Fig. 2C. Participants in the “up–down” class (blue) characterised by an initial increased rate followed by decreased biological ageing rate showed significantly greater improvements in quality of life compared with those in the “down–up” class (green) (mean ΔQoLIE = 18.8 versus 4.7; p = 0.007, Welch’s t-test). Horizontal lines indicate median values, and box limits represent interquartile ranges.

Next, we investigated whether clinical changes differed between participants grouped according to DunedinPACE trajectory patterns. No differences were observed between slow and fast agers defined by absolute DunedinPACE level (all p > 0.1). However, quality of life change differed significantly between the dynamic trajectory classes identified in Fig. 2C. Participants whose biological ageing rate increased initially and then slowed (“up–down”) experienced substantially greater improvements in QOLIE-89 scores compared with those showing the opposite pattern (“down–up”; mean ΔQOLIE = 18.8 versus 4.7; Welch’s t = 2.97, p = 0.007; Wilcoxon p = 0.018, Fig. 3). A non-parametric bootstrap analysis confirmed this effect, yielding an estimated mean difference of 14 points (95% BCa CI: 3.97–19.96), supporting the robustness of the association despite the smaller size of the up–down trajectory class. No corresponding between-cluster differences were observed for ΔBHB, ΔSeizure or ΔWeight (Supplementary Fig. S2), indicating that the improvement in quality of life was not explained by higher ketones, greater seizure reduction or weight loss.

## Discussion

This study provides evidence that adults with drug-resistant epilepsy exhibit substantial epigenetic age acceleration at baseline, as indicated by three cumulative DNAm clocks. These findings align with the chronic inflammatory, metabolic and psychosocial burden associated with epilepsy.^20^^,^^21^ Importantly, our results also show that modified KD therapy may transiently modulate the pace of epigenetic ageing, as captured by the DunedinPACE clock. While the mean group-level change in ageing rate was small and non-significant, participants showed substantial inter-individual heterogeneity in DunedinPACE trajectories over the 12-week intervention. Notably, the temporal pattern rather than the magnitude of DunedinPACE change was associated with improvements in quality of life. Together, these results highlight how cumulative and rate-based epigenetic clocks capture distinct biological dimensions of ageing, reflecting long-term disease burden and short-term physiological responses to modified KD therapy.

The Horvath, Hannum and Levine clocks quantify cumulative epigenetic age and reflect long-term molecular and physiological alterations that accumulate over the lifespan.^8^, ^9^, ^10^^,^^31^ In our cohort, Horvath and Hannum clocks showed pronounced age acceleration at baseline, consistent with evidence that chronic inflammatory, cardiometabolic, and neurological disorders often exhibit accelerated epigenetic ageing.^13^^,^^32^ Also at baseline, males had higher Horvath age acceleration, whereas Hannum and Levine showed no clear sex differences. This finding should be interpreted cautiously given the modest sample size and merits replication.

Epigenetic ageing has not been systematically characterised in epilepsy, and evidence in epilepsy cohorts is limited. Nonetheless, the chronic inflammatory and metabolic burden associated with long-standing, drug-resistant epilepsy provides a plausible biological basis for accelerated ageing signals. Together with widespread DNAm dysregulation and the burden of long-term anti-seizure medication exposure and chronic inflammation, our findings support the hypothesis that drug-resistant epilepsy may confer a substantial cumulative epigenetic ageing burden.

In contrast, the Levine clock yielded a younger-than-expected DNAm-predicted age in this cohort. This divergence likely reflects fundamental differences among epigenetic clocks. Horvath and Hannum were trained to predict chronological age from DNAm patterns across large datasets,^8^^,^^9^ whereas the Levine clock was developed to capture phenotypic ageing as a composite of clinical biomarkers linked to mortality risk and physiological state.^10^ Consequently, Levine estimates may be more sensitive to physiological parameters such inflammation, metabolic status, medication exposures and chronic stress. Divergence among clocks has been described across multiple clinical populations, indicating that different clocks measure different biological aspects.^13^^,^^31^

Epigenetic age acceleration was not associated with years lived with epilepsy, suggesting that the ageing signal is not simply driven by cumulative disease duration. Instead, accelerated epigenetic ageing may reflect broader systemic characteristics of chronic drug-resistant epilepsy, including persistent inflammatory signalling, metabolic alterations, or long-term pharmacological exposure.

A key observation is the contrast between the substantial baseline age acceleration and the slower-than-chronological ageing rate estimated by DunedinPACE. This divergence is consistent with the conceptual differences between cumulative epigenetic clocks and pace-of-ageing measure. Whereas cumulative clocks reflect long-term lifelong accumulated burden, DunedinPACE captures the current rate at which biological systems are ageing.^11^^,^^31^ Individuals may therefore carry a substantial cumulative biological burden due to long-standing epilepsy while simultaneously exhibiting a normal or even slowed pace of ageing if their current physiological state is relatively stable.

Although DunedinPACE showed no mean group-level change across the intervention, individual trajectories varied substantially. This heterogeneity is consistent with the rapid physiological remodelling known to occur during nutritional ketosis, which is also reflected in epigenetic changes.^27^ The metabolic changes induced by modified KD modulate several pathways implicated in biological ageing, including mitochondrial function, oxidative stress responses, inflammatory signalling, and chromatin regulation.^7^^,^^22^ Ketone bodies such as β-hydroxybutyrate can modulate mitochondrial efficiency, activate antioxidant and cellular stress-response pathways, regulate AMPK/mTOR signalling.^6^^,^^7^ Consistent with this, dietary and lifestyle interventions including KD and caloric restriction have been shown to modify epigenetic ageing trajectories in other populations.^14^^,^^16^^,^^17^

The level-based clusters reflected stable baseline differences in ageing rate that were not associated with clinical or metabolic outcomes during the intervention. In contrast, the trajectory-based clusters captured dynamic within-individual changes during therapy, revealing two dominant patterns: down–up (initial slowing, then rebound) and up–down (initial acceleration, then slowing). The “up–down” pattern was associated with the largest improvements in self-reported quality of life, independent of changes in seizure frequency, ketosis depth or weight. This finding suggests that DunedinPACE trajectories may capture a dimension of physiological adaptation or resilience not reflected in conventional clinical markers. Given the use of whole blood and the lack of a clear relationship between DunedinPACE dynamics and seizure outcomes in our data, the observed DunedinPACE changes during KD most plausibly reflect systemic metabolic and inflammatory adaptation to the intervention rather than epilepsy-specific mechanisms. More broadly, they highlight the potential value of rate-based epigenetic clocks for studying dynamic responses to metabolic interventions.

Several limitations should be considered. The sample size was modest, particularly for cluster-based analyses, and replication in larger cohorts will be necessary to confirm the trajectory patterns observed here. Accordingly, the trajectory clustering and between-cluster comparisons should be interpreted as exploratory and hypothesis-generating. The 12-week intervention period may also be insufficient to capture sustained changes in epigenetic ageing. In addition, DNAm was measured in whole blood rather than neuronal tissue and may not fully reflect brain-specific ageing processes. Although the association between DunedinPACE trajectories and quality-of-life improvement was robust, causality cannot be inferred. Nevertheless, the repeated-measures design integrating epigenetic, metabolic, and clinical data provides a framework for studying how metabolic interventions influence biological ageing dynamics.

In conclusion, adults with drug-resistant epilepsy appear to carry a substantial cumulative biological age burden, reflected by accelerated Horvath and Hannum epigenetic ages. In contrast, the Levine clock indicated a younger systemic biological age, underscoring the multidimensional nature of epigenetic ageing. During modified KD therapy, the pace-of-ageing biomarker DunedinPACE revealed heterogeneous changes in biological ageing rate that were associated with improvements in quality of life. These findings suggest that modulation of the current pace of biological ageing rather than cumulative ageing burden may contribute to the systemic effects of metabolic interventions. Rate-based epigenetic clocks may therefore represent promising tools for quantifying physiological responses to dietary and metabolic therapies.

## Contributors

KG conceived the current study idea, performed the statistical analyses, generated the visualisations, interpreted the data, drafted the manuscript, and contributed to writing, review and editing. MK contributed to conceptualisation, study design, funding acquisition, and data collection for the parent trial, and contributed to writing, review and editing of the current study. KON contributed to the design and conduct of the parent trial, clinical data interpretation, and writing, review and editing of the current study. KR contributed to the initial planning of the study, statistical methodology, data interpretation, and writing, review and editing. POI contributed to conceptualisation and supervision of the parent trial, and writing, review and editing of the current study. KKS conceived and supervised the current study, contributed to data interpretation and project administration, and contributed to writing, review and editing. KG and KKS accessed and verified the underlying data. All authors read and approved the final version of the manuscript.

## Data sharing statement

The raw data from this study are not available due to privacy and ethical restrictions related to the project approval and participant consent. Metadata generated in the study and code used in the analysis are available from the corresponding author upon reasonable request, within the constraints of the informed consent and applicable privacy regulations.

## Declaration of interests

MK has received two honoraria from Nutricia. KKS has received consulting fees and speaker honoraria from Roche and OrionPharma, reimbursement of travel and accommodation costs from Kolpharma, and sponsorships for arranging conferences from Desitin and Eisai AB paid to institution. KON has received lecture honoraria from Desitin and Eisai. The remaining authors declare no competing interests.
