# Supplementary material for: Epigenetic ageing is accelerated in drug-resistant epilepsy and dynamically modulated during ketogenic diet therapy
Source: eBioMedicine. 2026 Aug 22;131:106449. doi: 10.1016/j.ebiom.2026.106449 (PMC13524883; doi:10.1016/j.ebiom.2026.106449)
Supplement: Supplementary file 1 [file mmc1.docx]

**Web Appendix**

Supplementary material for: Epigenetic ageing is accelerated in drug-resistant epilepsy and dynamically modulated during modified ketogenic diet therapy

**Contents**

**Supplementary Table S1**

Baseline associations of epigenetic age acceleration

**Supplementary Table S2**

Within-person change in epigenetic age acceleration

**Supplementary Table S3**

Correlations between change in biological ageing rate and clinical outcomes

**Supplementary Figure S1**

Evaluation of *k*=1–4 to assess the choice of cluster number in the shape-based clustering.

**Supplementary Figure S2**

Comparison of additional clinical outcomes across DunedinPACE trajectory clusters

**Supplementary Table S1. Baseline associations of epigenetic age acceleration**

| **Model** | **Clock** | **β** | **SE** | **t** | **p value** | **FDR** |
| --- | --- | --- | --- | --- | --- | --- |
| Seizure frequency | Horvath | −0·005 | 0·013 | −0·406 | 0·687 | 0·800 |
| Seizure frequency | Hannum | 0·003 | 0·012 | 0·255 | 0·800 | 0·800 |
| Seizure frequency | Levine | −0·013 | 0·017 | −0·750 | 0·457 | 0·800 |
| Seizure frequency + sex | Horvath | −0·004 | 0·012 | −0·357 | 0·723 | 0·800 |
| Seizure frequency + sex | Hannum | 0·003 | 0·012 | 0·295 | 0·769 | 0·800 |
| Seizure frequency + sex | Levine | −0·012 | 0·017 | −0·727 | 0·471 | 0·800 |
| Epilepsy duration (years) | Horvath | −0·038 | 0·065 | −0·588 | 0·559 | 0·894 |
| Epilepsy duration (years) | Hannum | −0·051 | 0·059 | −0·872 | 0·387 | 0·894 |
| Epilepsy duration (years) | Levine | 0·011 | 0·084 | 0·133 | 0·894 | 0·894 |
| Epilepsy duration + sex | Horvath | −0·011 | 0·062 | −0·175 | 0·862 | 0·894 |
| Epilepsy duration + sex | Hannum | −0·040 | 0·059 | −0·679 | 0·500 | 0·894 |
| Epilepsy duration + sex | Levine | 0·020 | 0·086 | 0·235 | 0·815 | 0·894 |
| Sex (main effect) | Horvath | 4·563 | 1·474 | 3·095 | 0·003 | 0·010 |
| Sex (main effect) | Hannum | 2·037 | 1·420 | 1·434 | 0·158 | 0·236 |
| Sex (main effect) | Levine | 1·605 | 2·074 | 0·774 | 0·443 | 0·443 |

## **Supplementary Table S2. Within-person change in epigenetic age acceleration.**

| **Clock** | **Interval** | **t statistic** | **p value** | **Mean change in**  **age acceleration (years)** | **FDR** |
| --- | --- | --- | --- | --- | --- |
| Hannum | Four weeks vs baseline | 1·37 | 0·18 | 0·9 | 0·53 |
| Hannum | 12 weeks vs baseline | 2·8 | 0·0075 | 2·06 | 0·045 |
| Horvath | Four weeks vs baseline | 1·02 | 0·31 | 0·64 | 0·62 |
| Horvath | 12 weeks vs baseline | 0·81 | 0·42 | 0·58 | 0·63 |
| Levine | Four weeks vs baseline | 0·16 | 0·88 | 0·15 | 0·93 |
| Levine | 12 weeks vs baseline | 0·09 | 0·93 | 0·08 | 0·93 |

Mean change represents the difference between the specified timepoints.

FDR=Benjamini–Hochberg false discovery rate.

**Supplementary Table S3. Correlations between change in biological ageing rate and clinical/metabolic changes during the KD intervention.**

| **Clinical variable** | **Corrrelation** | **p value** | **FDR** |
| --- | --- | --- | --- |
| **Pearson** | | | |
| Δβ-hydroxybutyrate (mmol/L) | −0·291 | 0·275 | 0·856 |
| ΔWeight (kg) | 0·121 | 0·428 | 0·856 |
| ΔQOLIE-89 | 0·054 | 0·768 | 0·975 |
| ΔSeizure frequency | 0·005 | 0·975 | 0·975 |
| **Spearman** | | | |
| Δβ-hydroxybutyrate (mmol/L) | 0·101 | 0·479 | 0·487 |
| ΔWeight (kg) | 0·200 | 0·273 | 0·487 |
| ΔQOLIE-89 | 0·128 | 0·402 | 0·487 |
| ΔSeizure frequency | -0·188 | 0·487 | 0·487 |

Δ indicates change from baseline to 12 weeks.

QOLIE-89=Quality of Life in Epilepsy Inventory-89.

**Supplementary Figure S1. Internal validation of the number of DunedinPACE trajectory clusters.**

**A)** Elbow plot showing the total within-cluster sum of squares (WSS) for *k* = 1–4 clusters based on the shape-based clustering input (z-standardised ΔPACE 4 weeks–baseline and ΔPACE 12 weeks–4 weeks). **B)** Mean silhouette width for *k* = 2–4 computed from the same feature space. Together, the diagnostics support *k* = 2 as the most coherent and parsimonious solution for the trajectory-shape clustering in this dataset.

**Supplementary Figure S2. Comparison of additional clinical and metabolic outcomes across DunedinPACE trajectory clusters during KD treatment.**

Violin plots show changes in **A)** β-hydroxybutyrate, **B)** percentage change in seizure frequency, and **C)** body weight (kg) between the two DunedinPACE trajectory clusters (“down–up” and “up–down”). None of these variables differed significantly between clusters (all p > 0·10), indicating that the observed association with quality-of-life improvement was not explained by metabolic ketosis, seizure reduction, or weight loss. Horizontal lines indicate medians, boxes represent interquartile ranges, and white points denote group means.
